# Discussion before the Sixth Annual Meeting of the Ohio State Dental Society

**Published:** 1872-02

**Authors:** 


### Proceedings of Societies.

### DISCUSSION BEFORE THE SIXTH ANNUAL MEETING OF THE OHIO STATE DENTAL SOCIETY, DECEMBER 5–7, 1871.

*First Subject: Filling Teeth—Smooth-Pointed Pluggers.*

This subject being introduced, Dr. A. A. Blount, of Springfield, in speaking upon it, said it could be better demonstrated by clinics, but he would explain his method of using smooth-pointed fillers. He commenced the filling with a pellet of No. 6, 10, 20 or 30, as he saw fit, and took heavy foils and cut in strips from one eighth to one-sixteenth of an inch in width, and an inch to an inch and a half in length. Taking one end of the strip, he folded it back and forth, condensing its folds with a smooth point.

He claimed for smooth points an advantage in working up to the edge of a cavity in filling. The slightest blow on the edge of the enamel, with the serrated point, would be very apt to chip it off; with the smooth point there is no danger.

When the work of solidifying the gold is finished, the filling is complete It requires much less finishing, and can be done in less time, than where you fill with serrated points. No difference how thoroughly fillings may be made, with the serrated points, or Butler's instrument, which he thought as good of the kind as could be found, in a short time afterward the fillings, upon inspection, would be found to be full of small pits. Fillings made with a smooth point would not appear so, though examined a year or more afterward.

The question being asked if the slipping of the instrument would not injure the work, he replied that he did not have an instrument to slip on the foil, for the reason that he managed it so as to have a direct blow. If he wanted to spread the foil, he used an oval plugger. The blow with the oval plugger would be found to be more perfect than with a flat one, and that lateral expansion of the gold obtained, which has been sought for years, but never looked for in the instrument. It would be found, also, that the better the gold was burnished the better it would weld.

Dr. L. Buffett, of Cleveland, said if a direct force can be obtained, good fillings can be made; and another thing to be taken into consideration was, that there was always danger of the presence of moisture. Where this was the case, its influence would be shown sooner on a smooth or burnished surface than on a rough one.

The breath may be condensed and produce moisture, and affect the surface so that there may not be that adhesion that the operator may have supposed he had. Again, in some cavities it was very difficult to get a direct force, and there is a little slipping, and the gold will not adhere; for that reason he was a little afraid of the smooth-pointed pluggers. In some mouths it might be very successful, but in others he feared it would not be.

Dr. C. R. Taft, of Mansfield, said he had some experience with the smooth points. Some eight or ten months ago he had whetted off a plugger and went to filling with it, and found it packed as well, and, he thought, better than the serrated point. He had become better satisfied with it the longer he had used it, and did not think he would abandon it.

Dr. Blount, in answering the objection of Dr. Buffett, in regard to the appearance of moisture, said he thought it possible to keep the cavity perfectly dry, and that fillings made either with the serrated or smooth points, where there was moisture, would be imperfect, and there would not be complete adhesion. By the use of the rubber dam the moisture could be controlled.

Dr. N. W. Williams said he had been using smooth points since last May, at which time he heard Dr. Blount read a paper on the subject. He believed he was the first of his converts. He had but little faith in it until he went to Dr. Blount's office, and had him fill a tooth or two for him, and saw him fill others, when he became convinced. He had been using smooth points almost exclusively ever since, and believed that he had made better fillings than he ever did with serrated points. He believed the fillings were more easily finished, and that he could get in more gold. He thought if members of the Society would try this plan they would adopt it.

Dr. I. Williams said he had read, and carefully re-read, the article of Dr. Blount, and determined to test it. He gathered up a tooth or two, took them into his laboratory, prepared the cavities, and took some of Dr. Butler's instruments and ground off the points, screwed a tooth into the vise and went to work with the smooth points, carefully filling the cavity, and was astonished at the degree of adhesion there seemed to be

Upon examining the work with a strong magnifying glass, he could not see any of that porous condition that is usually manifest in fillings made with the serrated point. He tried again, with the same results, and was convinced that it was a success. He then prepared a number of instruments in this way a year and a half ago, and went to work upon the natural teeth in the mouth, using the rubber dam to prevent moisture, and was gratified with his success. He succeeded in packing the gold up against the walls of the tooth so perfectly that he felt certain the fluids of the mouth could not insinuate themselves between the fillings and the walls of the tooth. He had continued their use up to the present with the most gratifying results. He had not used them exclusively, but in the larger portion of his work

He was able to to get more gold into the cavity, because it was less porous.

Dr. C. R. Taft then read a paper he had prepared upon the subject of "Filling Teeth."

After the reading of this paper, the subject was further discussed.

Dr. E. J. Waye said he had experimented some with the smooth points. Although he could indorse the most that had been said, yet there were some difficulties in the way in the use of these pluggers. In small, deep cavities, which are easily reached, he thought more gold could be got in with the smooth points, but in larger cavities, difficult of access, he did not think there would be the same success, as the instrument would be inclined to slip if great care was not used.

Dr. Geo. Watt said he thought there was one thing to which enough attention had not been given; that was the compound motion employed in some fillings. If a straight instrument be placed upon the crown of a cavity of a lower molar, and the mallet be applied, the instrument descends obliquely; but suppose the operator is pressing in the opposite direction, and wishes to make a condensation on the center of it, the force would be oblique backward and downward so far as the mallet is concerned; but suppose he were making a pressure in the opposite direction with the instrument, and at the same time made the blow equal in that direction, the force would come upon it perpendicularly, and if he can have proper control of his malleter he can so signal as to have the blow about equal to the pressure he is making.

Trouble occasioned by moisture had been spoken of as a terrible thing, and so it was. The rubber dam which had been referred to was a good preventive of it, yet there was another instrument which had been longer before the profession, but perhaps used less; that was the warm air syringe. With that instrument absolute dryness could be obtained.

He did not believe, however, that there were ten of them in use; yet there was no instrument in dentistry, or out of dentistry, that does more perfectly what it pretends to do.

Dr. C. R. Butler thought the most desirable thing he knew in regard to packing gold was to know how it could be done most quickly and best. He knew nothing about the smooth point hobby, and regretted that if Dr. Blount had anything that was an improvement in the way of smooth points that facilitates the operation of filling teeth, that he hadn't brought them with him, that all might see them. The operation of filling should be done with as little loss of time as possible. Those who didn't use the rubber dam should never sleep after they go home until they get and learn how to use it. As a rule, he would use it in nine hundred and ninety-nine cases out of a thousand. That should be the first thing, for various reasons. In the first place, you have the case where you can control it better than where the patient is allowed to be stopping the operation by spitting. In the next place, it will save much time.

Again, where there is room to operate on a proximal cavity, if the teeth are very close, his practice was to draw in a piece of rubber for one day, then he would use compressed wood a day or two. Usually he would only use one piece, but in some cases would put in more than one. He preferred cottonwood compressed. When the patient comes to have an operation performed he would slip the wood out and put on the rubber dam. Everything should be ready as nearly as possible, so that when the patient comes in to take the chair the operator can go to work immediately.

Many fillings he did not finish up on the same day they were begun. If he feels that he is encroaching upon his patient's time, or upon the time of some other patient, he would let them retire and come again. The point he would particularly impress upon them was, that they should use as little time of their patients, and as little of their own time, as they could to get the best results.

Dr. C. R. Taft. How long do you have the patient wait before finishing the filling, and is not the tooth, in many cases, very sore, so as to give great pain when completing the filling?

Dr. Butler. The time necessary depends almost entirely upon the condition of the patient and the difficulty of filling the tooth. I have taken two or three days, to make it as comfortable to the patient as possible. If I were to do it rapidly I would take a strip of cottonwood and whittle it down to the thinness I desired to have the space, then, putting in the vise, bring it down gradually to the stiffness required. Wiping out the space by using the napkin, or anything to absorb the moisture, I would put in the wedge pretty firmly, so as not to strain, and then cut it off as near the ends as possible. Putting in the wedge to-day, on the day after to-morrow I would operate. Leaving the wedge in, I would adjust the rubber properly and drive the wedge gradually as far as necessary to give the required space. This will produce a slight numbness up toward the apex, and you can operate without much discomfort to the patient.

A member inquired what advantage the wooden wedge was over the rubber.

Dr. Watt replied that for one day the rubber would not do any harm, but it was objectionable, for the reason that it keeps up a continual dead strain. He was glad to see Dr. Butler following the rational method. Some might think if the rubber wedge gained so much the first twelve hours, it would be well to put in a thicker one and continue the process; but if this was done it would result in mischief. It sometimes produces periostitis.

Dr. J. Taft said he had followed the method of gradual separation for several years, but for the last few years he had been practicing prompt separation by wedges, with better success and more satisfaction than he ever had by gradual separation. He found but few cases, perhaps not one in three months, where he made the separation by gradual pressure. He generally made the separation at once, and the operation was performed immediately. Periostitis or soreness is very likely to occur when the pressure is made upon the teeth and allowed to remain a length of time. The principle stated by Dr. Watt in reference to this matter is correct; nevertheless, he believed it better to make prompt separation by wedging, perform the operation, relieve the teeth from the pressure, and let them go back to their normal position.

He had found, almost invariably, that more or less soreness of the periosteum was induced if the wedge was kept in twenty-four hours. Sometimes in two or three hours the teeth would be very sensitive from it. Often three or four teeth will become quite sensitive from the effects of the wedge, but by prompt wedging all this is avoided. Nature always makes efforts to accommodate herself to an altered condition. If the teeth are forcibly separated, there is an immediate effort of nature to relieve the point impinged upon, and will meet the necessity of the case sooner or later. He had seen a good many cases where the teeth were separated for two, three, four, or five days, in which they never approximated nearer together than they were left at the end of the operation, just for that reason—that nature accommodates herself to these new circumstances; but he didn't remember ever seeing any such occurrence from a prompt separation, followed by an immediate operation. He had heard of cases where persons had broken teeth by this method, but they may do violence by any plan, by roughness or awkwardness in the operation; but when properly done there would be no bad results. The greatest pain of the patient is upon the first introduction of the wedges, but you can obtund the sensitiveness by driving the wedges so that there will be scarcely any pain.

Dr. C. R. Butler regarded the subject as of great importance. He was in favor of immediate separation of the teeth where practicable, but there were circumstances where it could not be done with safety. He supposed some cases where it would be difficult to pack the gold for the lack of sufficient room. If, in the case of a lateral incisor, the wedge was driven so as to open a space immediately, he thought there would be risk of fracturing the enamel, and so impair the strength of the tooth. In all cases he believed it best to gain the necessary space as quickly as it could be done safely. He never saw, in his experience. any case where the separation remained as left by the wedges. Where such was the case he thought it must be the result of improper management. Neither had he any case of periostitis to arise from this method. In either case the dentist must be governed by the age and condition of the patient. What may be best in one case would not do in another.

Dr. B. T. Spellman said he formerly wedged entirely by the slow process, but had very seldom practiced it in the last few years; he had resorted to it for a few hours, perhaps from the morning until the afternoon. The physiological change which takes place by the slow process of wedging, every dentist should avoid. In the slow process with the wooden wedge, the force is expended upon one side of the transverse process, and the result is that the capillaries are obliterated and absorption takes place, and on the other side of the process hypertrophy results. These are very serious objections to the slow process of wedging, and should be avoided.

QUIZ EXERCISE.

Quiz was conducted by Dr. Geo. Watt.

Question. Is there any reason why inflammation in the socket of the tooth is more painful than in any other part?

Answer, by Dr. B. T. Spellman. The periosteum being the seat of inflammation, and being bound by the structure on both sides, it is more easily injured by impinging; and there being an unyielding boundary to the periosteum, makes it more sensitive.

Q. What are the ordinary phenomena of inflammation?

A. by Dr. J. Taft. In the ordinary soft tissues, heat, redness, swelling and pain. The same thing is true of the periosteum to a certain extent; perhaps all the phenomena attending inflammation in soft tissues may exist in inflammation of the periosteum.

Q. Why is pain one of the phenomena of inflammation ordinarily?

A. by Dr. I. Williams. There is a pressure upon the nerves, owing to the increased flow of blood to the part; that is, pain results from the increased flow of blood, causing pressure upon the nervous fibres.

Q. Is it due to the increase of blood in the vessels?

A. by Dr. J. F. McGinnis. He should think it was, in a great measure, owing to the influx of blood to the parts.

Q. Swelling is one of the ordinary phenomena of inflammation; do you then suppose that the vessels of the periosteum enlarge when it is inflamed?

A. by Dr. Decamp. Yes, sir, I think they do; that is, thickened, if you call that swelling. I do not know what else it would be.

Q. What is the consequence of that swelling with regard to the teeth?

A. by a member. The consequence necessarily is pain, because as soon as the membrane commences to swell, or, being congested, undue pressure is created upon the nerves, the membrane being exceedingly vascular. I regard that as the reason why the membrane is more painful than inflammation at any other point. By extracting and removing the root of the tooth, the vessels have room to expand without undue pressure, and the pain will cease.

Q. Does the tooth change position and protrude from the socket when inflamed?

A. by Dr. Kelsey. Of course, while there is a thickened periosteum, the tooth stands partly out of the socket. It is not all imagination that the tooth has started up ; it really is thrown slightly out of the socket.

Q. Is there any tissue, or any constituents of the blood, that becomes extravascular ?

A. I suppose some may be thrown out in passing through the vessels.

Q. Suppose, from dental periostitis, suppuration takes place, what precedes suppuration ?

A. by Dr. Clippenger. I would describe it as an inflammation or engorgement of the vessels until the circulation stops. When suppuration is found in the socket there is usually a sac formed.

Q. Will you give your opinion how that sac is formed?

A. It is of a fibrous nature, composed of fibrous tissues.

Q. Is that sac merely a distended portion of the periosteum, or is it formed of effused lymph matter that is thrown out?

A. by Dr. I. Williams. It is maintained by some that it is formed by the expanding of the periosteum. I think it is not an expansion of the periosteum, but mainly from the effusion of the lymph.

Q. If you have a case of acute alveolar abscess, not yet discharging through the gum, and not ready to make an artificial opening, tell us what would be your treatment?

A. by Dr. C. R. Butler. As I understand it, you mean a case of acute alveolar abscess, where pus is formed, but has no exit. I think the best way to master the trouble would be simply to make an incision—tap it and get rid of the pus formed, as quickly as possible, so that the parts surrounding it would not be much involved.

Q. Suppose a patient comes to you with a tooth in such a condition that you suppose it may be saved and made valuable, but chronic alveolar abscess takes place, would you try to save the tooth, or extract it?

A. by a member. I would treat it so as to heal up the abscess in the shortest time possible, and would fill up the tooth with some substance that would exclude the moisture and gaseous matter, and fill the tooth. If it was an obstinate case, I would make an opening; but with ordinary cases it could be made through the opening of the tooth. In filling the roots, I think non-conducting substances better than metal.

Q. What would you regard as favorable or unfavorable symptoms, in reference to the treatment, in cases of this kind?

A. by Dr. L. Buffett. With patients of a scrofulous tendency we could not expect as good results. If the patient be healthy, with no such taint, we expect to save the teeth. Age, also, would have some effect. We expect better results with middle aged or young persons than with older persons whose powers were weakened by age.

Q. Would you depend upon constitutional treatment in cases of periostial irritation, or would your treatment be almost entirely local?

A. Many cases are local, to a great extent, and may be easily treated locally.

Q. Suppose you found a case of alveolar abscess resisting treatment, and continuing to discharge in spite of your treatment, are there any circumstances where you would let the tooth remain? If so, under what circumstances?

A. If I found the tooth resisting all treatment—that is, both constitutional and local treatment—yet I can not think of any such case ever coming under my observation—I would feel obliged to remove the tooth, unless it produced no soreness or inconvenience to the patient.

Q. How are the pus globules formed, and where do they come from?

A. by Dr. J. Taft. I really want more light upon that subject. I know there are various views entertained. One is, that they are just like the white corpuscles of the blood, and are a multiplication of these. That is as far as I have known anybody to go on that hypothesis. That is not at all satisfactory; it does not tell us how that multiplication takes place. My impression is, however, that pus corpuscles are formed by the perverted use of the pabulum that is prepared to nourish the tissues in which the change takes place. By disease, the action is going on in that part, which perverts this from its original use, and it takes on this form.

### FILLING TEETH.

The subject of filling teeth was then resumed.

Dr. L. Buffett said he had been asked whether, supposing three or four incisors were to be operated upon, and wedging were necessary, he would gain the space between all of them at the same time. He regarded that as improper. He would have the patient to understand that it was necessary to gain space between them, in order to have room to work, and would open one space, and then, after the lapse of a few days, would open another.

Dr. Geo. Watt remarked that it seemed necessary sometimes, after getting teeth ready to fill, to postpone the work because of some change going on in the constitution of the patient, or some disease that may impair the vitality of the periosteum; but it was advisable, and had been his practice for the last few years, to fill in such cases with some non-conducting substance. He usually took artificial dentine, gutta percha, or Hill's stopping, and filled with that. After getting the cavity filled, as nearly up to the apex as possible, he finished the filling with gold. Dr. Hunt used to remark, that it was best sometimes to get the teeth ready to fill, but not fill them. He would say so too, if he was sure the condition of the patient would not be changed so as to make it not best to do so.

Dr. C. R. Butler said he used oxychloride in such cases. He thought the practice of using cotton was a very bad one. He introduced the oxychloride with a bit of wood. Taking a piece of hickory and filling up as far as he could, he then took gold. He could thus fill the canal more perfectly than if done entirely with gold. The oxychloride will fill it more perfectly—will exclude all the gases and the ingress of any foreign substances. He agreed with Dr. Watt, that a non-conductor was much the best, and has a salutary effect upon the structure.

Dr. W. M. Herriott said, in pursuing the practice introduced by this Society in past years, he had found, as an effect of the use of oxychloride of zinc upon the tissues, upon cutting in a year after it had been used, that the nerve was dead. If the destruction of the life of the nerve was desirable, he thought it would have a salutary effect. It might be as Dr. Butler said, that this chloride of zinc had a good effect upon the periosteum of the socket; but, if so, it is contrary to the changes he had observed as to its action upon the life of the nerve.

Dr. Spellman. I would ask if in these cases you found an abscess?

Dr. Herriott. I have found cases where there were not abscesses, in which the nerves were dead.

Q. Was the nerve in a state of disintegration?

Dr. Herriott. The whole pulp tissue of the tooth was dead, as appeared to me, by the action of the chloride of zinc.

Dr. Butler said there were gentlemen in this Society, and in others, who have heard it said that it would save the tooth; but when they say it will save the pulp in a live state, it is not true. There are extreme cases where it might, but in a majority of cases, according to his observation, it would not do it. He related a case that had come under his observation, where the pulp remained alive over a year where the oxychloride filling had been used.

Dr. H. A. Smith, in referring to a remark that had been made recommending holding the gold above the flame of the lamp in annealing it, said he would not advise such a plan. He held his in the hottest part of the flame. He thought that Dr. Blount deserved a great deal of praise for his energy and zeal in regard to smooth points.

Some men thought that absolute dryness was impossible; he, however, could conceive many cases where it was possible and highly profitable. He had found the hot air pump very useful, in the oxychloride fillings, to throw a jet of hot air upon them. He considered it radically wrong to use crystal gold with smooth-pointed pluggers.

Dr. Geo. Watt said when the gold, in heating, was held above the flame it was in a vapor of water, or in carbonic acid gas. He had seen persons trying to dry instruments in the same way, and had seen the water deposit on the steel. That was not the place to anneal gold. He had tried the hot-air syringe several hundreds of times, and never failed of success with its use. Dr. Taft and he had formerly filled with smooth points, but as soon as serrated points came into use they thought they could fill better with them. He knew that Dr. Williams, who had been filling with smooth points for the last few months, had made good fillings. For his part, he would dislike to lay aside serrated points.

Dr. Herriott. In filling with gold, are the parts of the gold held together as two pieces of glass with perfectly polished surfaces would be, or by the uniting and interlocking of the crystals?

Dr. Watt. When properly done, it is held together as two pieces of iron are when heated to a bright red heat—just as welding, when properly done.

Q. What holds the particles of iron together?

Dr. Watt. Cohesive attraction.

### DISCUSSION ON DR. TAFT'S RESOLUTION RELATING TO THE APPOINTMENT OF NORMAL CLASS TEACHERS OR CLINICAL OPERATORS.

Dr. Taft, upon offering the resolution, said that his reason for presenting it was that there were frequent calls made and desires expressed for such assistance as contemplated in the resolution, both in our own State and elsewhere. He would have this work inaugurated in our own State, believed much good could be accomplished by it, and the Ohio State Dental Society may take the initiative steps in this matter and gain whatever of prestige there may attach to it.

Many things, which may be considered small points in the profession, when their influence is taken into account, are great ones. The application of the rubber dam has been one of these. Perhaps not one in ten or twenty in our State understands properly the use of that appliance. If its proper use could be shown and understood by the profession in our State, it would almost revolutionize their practice. So with regard to many other things.

These teachers should organize local societies, and do all the work possible for the elevation of the profession wherever they go, but should especially make clinical demonstrations. If men were thus sent out under the sanction of this Society, they would be received with entire confidence, and their labors could not fail to be beneficial.

Dr. C R. Butler concurred in the views expressed by Dr. Taft upon this subject.

Dr. Geo. Watt thought this proposition made provision for clinics in their proper place. In large societies like this, it is very unsatisfactory to attempt it, and occasions a loss of time.

Dr. F. H. Rehwinkle was decidedly in favor of the motion, for the reasons that had been so well stated already. This Society ought to take this step. He hoped to see it stand number one in the United States and Europe. The actions of this Society are watched with particular interest, and the opinions it expresses on matters connected with the profession have great weight in every direction. The influences of the step proposed in the resolution would be felt everywhere.

After a few remarks by Drs. Berry and C. R. Taft, the resolution was unanimously adopted, and the Society adjourned until 2 P. M.

### TREATMENT OF DENTAL IRREGULARITIES.

The first subject taken up at the afternoon session was the treatment of dental irregularities.

Dr. Berry said that in this country the irregularity of the teeth is a great evil, as we have all the nations of the earth mixed up with us. The broad jaws and large mouth, the little jaws and small mouth; some with small jaws and large teeth, and others with large mouth and small teeth. And it is often difficult to make people appreciate the difficulties attending such irregularities. There is a lamentable lack of knowledge on this subject on the part of the public, and the subject, he thought, could be profitably discussed.

Dr. Watt said there was no one thing that was such a standing reproach to the dental profession as the state of mind with regard to dental irregularities and the methods of treatment by the profession generally. He remarked that the people about Xenia had confidence in filling teeth, and knew what it meant, and some had confidence in the infernal rubber, and a good many appreciated artificial teeth; but they are not awake, even there, to this subject, and will express their surprise that you propose to charge them twenty-five dollars for bringing the central incisors to the proper position when they shut on the inside of the lower ones This was about the simplest kind of case they were called upon to treat. He considered it a disgrace to the profession that a better state of things in this respect did not exist. He took a full share of this disgrace upon himself. It was not because cases of irregularity had not been properly treated. As far back as the winter of 1842–3, Dr. J. Taft regulated one of the most difficult cases of irregularities he had ever saw, and with complete success. He believed they had nearly spoiled the people by allowing them to expect that such work will be done almost for nothing. They have the idea that it must be done cheap. They must be educated upon this point, and made to understand that it involves the preservation of the teeth; that human beauty is involved, and that these defects can and should be removed. Until the public mind is educated properly on this subject, much in this direction could not be accomplished. The profession has many difficulties to contend with in this matter. One of these difficulties is artificial work. Artificial dentures have become so cheap, and people think they look so nice, that they are ready to have these irregular teeth extracted and artificial ones take their place. Propose to them to regulate the natural teeth in a difficult case for fifty dollars, and they will say: "Oh, I can get a new set of teeth for twenty-five dollars." If people were made aware that the extraction of the natural teeth shortens life, this difficulty might be removed to a great extent. Dentists should refuse to do work for those who come to them, unless they are willing it should be done in a proper manner.

He then related a case that occurred during his practice in Cincinnati. A young lady came to him to have thirty-two natural teeth extracted. The teeth were sound and beautiful, the young lady about thirty years of age. All the trouble was, her beau thought her front teeth were a little more prominent than they ought to be. He refused to extract them, and told her if they were to be extracted, it would perhaps shorten her life ten years, and probably more, and would mar her beauty besides. He told her that her natural teeth could be regulated so that the slight irregularity that existed would be overcome, but she did not consent to this, and went away. The next day the young lady and her mother came back, the mother insisting that the teeth should be extracted, and that he should not dictate to them what they should do, but should do what they desired. He still refused, and talked pretty sharply to them, threatening to arrest them if they employed any one to extract those thirty-two sound teeth.

Dr. Rosson related a couple of cases of irregularity he had treated, in which he had thought it impossible to do so without extracting two teeth in each case, but thought, with his present knowledge on the subject, that he could have saved them.

Dr. Herriott described a case of irregularity he had treated. The patient was a girl of thirteen years of age, in whose mouth the right canine tooth was nearly half an inch inside of where it should be, striking entirely inside of the lower tooth. The lateral incisors were apparently out very much, but the position they occupied was only apparent—that is, so far as they related to the other teeth and the external appearance of the mouth. The appearance was owing to the eye tooth being in so far. He took a gold plate and made a clasp upon it, which he passed back of this eye tooth, and made a gold spring and soldered on the inside of the clasp, and let it come against the tooth with a little pressure. This appliance was worn about six weeks or two months. That eye tooth was now in its proper place, and the front teeth did not appear to be out as they were before, the teeth in the mouth appearing to be well arranged.

Dr. Butler thought they should all understand this matter, so that they could convince the people that the irregularities of their children's teeth could be corrected, just as much as that the teeth can be filled. The people did not understand this, and the profession are to blame for at least a portion of this want of confidence in this respect.

Dr. Sedgewick thought this a subject which had been much neglected heretofore, and many in the profession did not know how to treat cases of irregularity. There are so many different kinds of irregularities presented to them, in so many different phases, that it is not possible to lay down any set of rules for their treatment. A case of frequent irregularity is, where the canines stand outside of the arch. Many cases come under their notice where the cuspids are gone, and if the patient is asked if they were decayed, he will tell you he had a tusk and had it pulled out. Many of these cases might be brought down by a simple ligature. The most difficult cases of irregularity that came under their notice, he apprehended, was where the upper incisors shut inside of the lower ones. He had treated some cases of this kind, but he did not like to undertake them, as they were tedious and troublesome to manage; but the duty devolved upon them; they need not expect to get sufficient pecuniary reward to compensate them for all their trouble, and the time that must be spent and the inconvenience attending such cases.

Dr. Watt said he had treated some hundreds of cases of irregularities, especially of the canine teeth. The principle of rest and labor, which he advocated on the preceding day, he thought was a correct one. The idea that there must be a constant pressure, or no good could be done, was incorrect. Where the central incisors shut inside of the lower teeth, it is unnecessary to have any appliance to keep up a constant pressure. It is better not to have constant pressure. A stick like a pencil, with a notch in it, can be made effectual in such cases by the patient using it to obtain the necessary pressure, and, after they get in the habit of using it, the soreness experienced will be when it is not pressing upon the teeth. The patient can remove this stick before eating or retiring to rest at night. He had regulated many cases by this simple contrivance. If they would bear in mind that constant pressure is deleterious rather than advantageous, they would be better able to succeed. He thought that in most cases they were inclined to use apparatus that was too complex. Truth and simplicity are twin sisters.

Dr. L. Buffett thought few of them had the faculty of regulating many cases like Dr. Watt. He believed, as Dr. Watt said, that the true principle in applying the force required was, after it was applied for awhile, to ease it up, and not try to obtain constant pressure. He explained his use of a silk band, in some cases of irregularity, which he had found successful. One reason of failure in some cases is, that the operator does not make up his mind with the definite object in view which he desires to accomplish.

Dr. Watt remarked that one trouble was that operators tried to do too much at a time. He tried to get rid of all springs and complex appliances in the mouth he possibly could.

Dr. Blount related a case of irregularity which he had recently corrected. The arch in the mouth of the patient, both above and below, was very much contracted from the bicuspids forward. The two central incisors turned in together, and the proximal central incisor turned out. He made a plate, with little uprights, behind each of the bicuspids, and took the plan Dr. Watt suggested, of putting wooden wedges in, pressing it up, and leaving them in for a few days, until the soreness had subsided. He kept this up until the upper arch had expanded sufficiently, paying no attention to the lower arch. He did not want the patient to wear the plate for any length of time, for fear of injuring the teeth where the plate pressed against them. He made a little gold staple, or what is called a dog, and used that to facilitate the work. The central incisors, having cavities in them, he filled them. It had been about two months since the operation had been performed, and those teeth were straight, and the lower teeth were gradually coming in place themselves, and, he thought, in a few months would be entirely straight. The principle stated by Dr. Watt, of allowing rest, and not making a persistent pressure, he had learned from him in some remarks he had made before the Miami Valley Association, and had acted upon it ever since, he believed, with profit.

Dr. J. H. Warner thought the principle advocated by Dr. Watt, of labor and rest, was a very important one to the profession, and might be applied with much benefit. He believed by that plan, a large amount of the annoyance which arises from the correction of irregularities could be obviated. The strain which is often applied, he had no doubt, was many times too long continued.

Dr. Watt remarked further, upon this subject, that usually the plan he had suggested would make enough pressure the first day to produce such a condition of the periosteum that it becomes unpleasant to press in any other direction. Suppose the case of an upper incisor shutting inside of the lower ones, and the pressure to be made during the day and stopping at night, and there would be, the first night usually, such a condition that there would be uneasiness if the tooth is pressed back again. That uneasiness will prevent the patient from shutting the teeth tight during sleep; hence, what is gained during the day is not lost at night.

### DISCUSSION UPON THE "RUBBER QUESTION."

Dr. Berry referred to the employment of Colonel Fisher as a legal adviser in the suit in regard to this matter. He had told them they would be beaten, but no doubt, under the suit which covers the patent, they had beaten the company. They went into the fight contemplating the time when the Goodyear patent would expire, and did not suppose that the company would buy Congress again. He had no doubt the rubber company was able to buy Congress and get another extension. He hardly supposed they would, though they might do it. He supposed they would only have the Cummings patent to confront them. The question was, shall they wait a year longer, and submit to the imposition of the rubber company? They should consider what it was best to do in the future in regard to the matter.

Dr. Rehwinkle said he had thought upon the subject a good deal, and thought it advisable to call the attention of the State Society to it in his annual address last year. He said after the judgment was rendered in favor of the Boston Vulcanite Company, in the case of Webber against the company, the matter looked as if it was almost hopeless; but, notwithstanding all that, the profession in Ohio took the initiatory steps to defeat the Cummings patent; but, in order to do this, they had to climb over the Goodyear patent. The time for the Goodyear patent would expire in 1872. It was impossible to tell whether it would be extended or not; but whether it is or not, they may come and demand fifty or seventy-five dollars for a license to use the Cummings patent. From all he had heard and known in regard to the matter, he did not believe the Cummings patent could hold out in another contest, before another United States Judge. He thought they were coming where they could reach the Cummings patent, and thought it advisable, just, and right for them to stick together in this matter, whatever their opinions might be in reference to the merits or demerits of rubber. They could not ignore the fact that it was used extensively, and he was not prepared to say that rubber should not be taken into the mouth. If they were prepared to say they would have nothing more to do with rubber, that would settle the question; but there were those among them that did not feel willing to do so. If anything was done, they should work together unanimously and fight against this monopoly. He suggested the appointment of a committee of persons who were thoroughly posted on the subject, to investigate it further and do what they deemed best in the case.

Dr. Rosson thought the time had not yet come to fight the Cummings patent.

Dr. Herriott desired to know when it would come. If any action was to be taken in the matter, it should be done now, as it would be too late by the time the Society would meet next year. He did not use rubber at all, and did not know that he ever would; but he knew there were those in the profession who use it, and feel compelled to use it. For that reason he thought the matter should receive the attention of the Society, and measures be taken to afford such persons relief. Something should be done, or the subject entirely abandoned. He had the opinion of patent-right lawyers in regard to their liability in using rubber under the Cummings patent, and though the agents have led dentists to believe they were compelled to pay license under it, there was nothing of it.

Dr. Butler apprehended there was nothing binding in signing the license under the Cummings patent, unless it be what is called the thousand dollar bond. But it was believed that that little clause did not amount to anything, and would not be recognized by the courts. He knew some who had signed it under protest, and thought perhaps all had done so.

Dr. W. P. Horton said if the records of the Society from 1866 to 1869 were examined, a tolerably fair status of this matter would be found. In defending the profession against the Goodyear Company, he was sorry to say that less than one hundred out of the four hundred dentists in the State aided in the prosecution of this matter. In the fall of 1869, after this had been going on three years, Mr. Bacon came out here after he heard of Judge Leavitt's decision against certain gentlemen in Cincinnati, to see about it. The Goodyear patent and the Cummings patent were separate considerations, and the Goodyear the only one litigated in the State of Ohio at that time. Everything looking toward the Cummings patent was withdrawn, and the suit commenced on the Goodyear patent, and on that alone. Col. Fisher had told them that he could not successfully contest that, and that judgment would be rendered against them, as it was in the original question. Col. Fisher urged this in his argument, and made the point that the remedy was in law and not in equity. He had no doubt in his mind but that the Cummings patent was an infringement on the Goodyear patent. This matter must be looked in the face, and hence he proposed the appointment of a committee to report the next day what action should be taken, and the reasons for it. If any action was to be taken, it was time now to do so.

(To be continued.)

